# Neuroeducation, Motivation, and Physical Activity in Students of Physical Education

**DOI:** 10.3390/ijerph18052622

**Published:** 2021-03-05

**Authors:** Antonio Baena-Extremera, Pedro Jesús Ruiz-Montero, David Hortigüela-Alcalá

**Affiliations:** 1 Department of Education Sciences, Faculty of Education, University Campus of Cartuja, University of Granada, s/n, 18071 Granada, Spain; abaenaextrem@ugr.es; 2 Department of Physical Education and Sport, Faculty of Education and Sport Sciences, Campus of Melilla, University of Granada, 52071 Melilla, Spain; 3 Department of Specific Didactics, Faculty of Education, University of Burgos, 09001 Burgos, Spain; dhortiguela@ubu.es

## 1. Introduction

In recent years, neuroscience and neurodidactics have demonstrated significant progress in improving the teaching and learning process for various subjects, such as physical education. As a reflection of this progress, very interesting studies have appeared in the scientific literature concerning the motivation processes of students and the influence of physical activity on brain processes and structures. In this sense, some papers have explained how physical activity can affect both the psychological aspects related to learning, as well as the neuroscientific aspects related to the cognitive functions of students. In addition, recent work has shown that many of these variables can be addressed by the teacher or the student, and a close relationship was found between both the development and evolution of the physical education class and its future school performance.

This Special Issue was proposed to collect the most recent research regarding the neuroeducation, motivation, and physical activity in students of physical education, focusing primarily on the analysis of the variables that can affect students and teachers within the didactics and learning-teaching of Physical Education.

## 2. The Studies Included

We received a total of 24 submissions, of which 20 were finally accepted in this issue, with a deadline of December 2020. As readers will see, most of the accepted publications used cross-sectional methodologies, but there are papers with the multiple mediation model, multi-level analysis, manuscripts using EEG, or with systematic reviews and meta-analysis. The majority of the studies were conducted in Spain, although some studies were conducted in other countries.

Presented in chronological order of publication within 2020, this Special Issue includes the papers described below.

The first manuscript, by López-Belmonte et al. [1], addresses gamification and escape rooms as a methodological tool to generate activation in students. Using a control group and two experimental groups, through a mixed research methodology, the results showed how the groups that received gamification obtained better results in the variables of motivation, teamwork, and commitment to the tasks. Thus, gamification could be an interesting didactic methodology with an attractive factor for students. The second manuscript, by Barba-Martín et al. [2], is a systematic review about teaching games in physical education (TGPE) between 2014 and 2019. Four databases were used to select manuscripts related to TGPE, with a total of 12 articles which met the eight criteria selected. Motor and cognitive learning were the most frequently assessed between all articles of this study. The main finding was that TGPE may produce positive effects in the primary and secondary physical education lessons. The next manuscript was by Ureña-Lopera et al. [3], where the main aim was to analyze the motivation of soccer players of developmental age during training time (baseline) and precompetition time. Different categories, sport success, playing position, and relations of the motivation dimensions with the academic performance were taken into account in this study. In conclusion, under 16-year-old soccer players showed lower levels of motivation before the competition. The fourth research by Moreno-Guerrero et al. [4] deals with augmented reality within the subject of physical education. The results show how high school students (*n* = 140) who experienced these experiences obtained greater motivation and learning in the subject, especially in terms of spatial orientation. The methodology was quantified by a design post-test. The fifth manuscript, by Fierro-Suero et al. [5], validated the Supporting Basic Psychological Needs-4 (SBPN-4) questionnaire in physical education, which included the novelty support factor. In addition, they tested the mediation model to confirm the effect of novelty support in relation to basic psychological needs and intrinsic motivation. The sample was composed by 723 students (mean age = 13.3 years). In conclusion, the SBPN-4 was shown to be a valid and reliable tool.

The sixth research, conducted by Romero-Rodríguez et al. [6], addresses the influence of smartphone addiction and intensity of Instagram use on the self-esteem of physical education students. A cross-sectional intervention was performed with 385 undergraduate students. The results showed that gender and age were factors influencing problematic smartphone use, with a significant positive correlation between smartphone addiction and intensity of Instagram use. The influence of smartphone addiction on students’ self-esteem was also highlighted. The seventh manuscript, by Carlos Fernández-Espínola et al. [7], reviewed the effects of cooperative learning interventions on intrinsic motivation in physical education students, demonstrating the relevance of factors, such as the duration of the program and the age of the participants, in order for cooperative learning to be carried out effectively. This manuscript followed the PRISMA guidelines to conduct a systematic review and meta-analysis. In addition, PEDro scale was used to assess the risk of bias and evaluate the quality of the evidence. The eighth manuscript, by Ferriz-Valero et al. [8], is the second study about gamification in this Special Issue. Analyses of the effects of gamification on the motivations and academic performance of university students (*n* = 127; experimental group = 62, control group = 65), show that its application in physical education led to an increase in students’ academic performance. The ninth manuscript, by Sicilia et al. [9], analyzed the relationship between parental and peer autonomy support and intention to exercise in adolescents (*n* = 428) between 13 and 19 years old, also considering the mediating role of attitude, control, subjective norms, and descriptive norms. The results showed that perceived parental autonomy support was positively and statistically significantly associated with exercise intention. Main findings suggest that subjective/descriptive norms and perceptions of autonomy could be more important than other forms of social influence in the exercise intention’s preference of adolescents.

The tenth research by Petisco-Rodriguez et al. [10] analyzed whether female athletes, in particular gymnasts and female football players, have more eating problems compared to non-athletes, looking at variables such as anxiety, self-esteem, and perfectionism. The results showed how non-athletic adolescent girls showed more disturbed eating behaviors and thoughts than adolescent girls in aesthetic sports. The eleventh investigation, by García-Monge et al. [11], analyzed differences in brain activity in various types of throwing games by making encephalographic recordings. Three conditions of throwing games were compared, looking for significant differences (single throw, throw to goal, and simultaneous throw with another player). The results found significant differences especially in the high beta oscillations (22–30 Hz). The “goal” and “simultaneous” throwing conditions showed significantly higher values than those shown for throwing without an opponent. The twelfth study is related to gender, physical self-perception (PSP) and overall physical fitness (OPF) in secondary school students (*n* = 41 boys and 44 girls) between 12 and 17 years. Ruiz-Montero et al. [12] were the authors, and they tried to compare the PSP and OPF between gender and body mass index status in the participants. A multiple mediation analysis was performed with significant results (*p* = 0.05). The findings showed the girls as a risk group because they felt lower PSP and OPF than boys. The thirteenth manuscript, by Martínez-Díaz et al. [13], aimed to determine the effects of an acute High Intensity Interval Trainig (HIIT) session on neurocognitive and stress-related biomarkers and their association with working memory capacity in healthy young adults. Significant post-exercise increases in circulating levels of plasma brain-derived neurotrophic factor and cortisol were observed, coinciding with increased performance on the digit span test. The fourteenth manuscript, by Coterón et al. [14], compared the influence of teachers’ satisfaction (*n* = 29) of basic psychological needs and frustration of basic psychological needs on students’ behavioral (*n* = 644) engagement. The results revealed that, although teachers’ autonomy satisfaction might be significant in explaining students’ engagement, need frustration might be a better predictor of this outcome. The fifteenth study, by Chiva-Bartoll et al. [15], analyzes the importance of the application of service learning in the initial training of Physical Education (PE) teachers, showing significant improvements in the dimensions of social self-fulfillment and problem-solving self-efficacy.

The H’mida et al. [16] study was the next manuscript included in this Special Issue. The aim of this study was to compare the effectiveness of a video and different pictures (simultaneous-permanent, sequential-transient, and sequential-permanent) on complex judo skill among novice young adults (*n* = 104). The results showed that the sequential-permanent picture was the most effective presentation. The seventeenth manuscript, by Ureña et al. [17], studied the self-regulation (SR) in pre-schoolers (*n* = 49) between four and five years as strong predictor of mental health and wellbeing. They were submitted to classroom movement breaks (CMB) of fifteen minutes with different difficulty levels. Pre-schoolers were stimulated only with CMB according to their cognition.

The three next studies were published in 2021. The eighteenth manuscript, by Manzano-León [18], is about the role of playful learning strategies. Gamification, game-based learning, and escape rooms are methodologies used with university students (*n* = 450) between 18 and 55 years in the present study. A confirmatory factor analysis was performed to analyze the motivation for playful learning strategies used in this study, and therefore, in the Spanish higher education context. The nineteenth manuscript, by Liu and Lipowski [19], addressed sports gamification on university students’ (*n* = 150) learning motivation and performances in physical education by tennis practice. The total samples was divided in two groups (control and experimental group). Students of the experimental group increased their intrinsic motivation and introjected regulation. Finally, the last manuscript is a study with 303 physical education students by Crisol Moya and Caurcel Cara [20]. They incorporated innovative methodologies as pedagogic models necessary in the training of students. A structural equation model for analyzing predictive relations between methodological factors (organizing modalities, methodological approaches, and evaluation systems) were used. Positive relations between those three factors were found, and indicated that perception and opinion of the physical education students took a special role in the improvement of learning and the methodologies used.

## 3. Conclusions

Of twenty manuscripts, four of them used gamification in their learning methods with students in the physical education field. Most of them were published in 2020 (*n* = 17), and majority of participants were university students. This Special Issue proves the importance and usefulness of the neuroeducation, motivation, and practice of physical activity on students and general population. However, most of the results are based on the physical education field. Two systematic reviews were included in the present Special Issue. The rest of manuscript used quantitative methods (*n* = 17), and only one used a mixed method (quantitative and qualitative research). We could conclude that the quantitative method in the psychologic research is most frequently used if we observe the analysis performed in most of the manuscripts. Moreover, most of the studies were performed with Spanish participants in pre-school, primary, secondary, and university students, except for two manuscripts that were performed with Chinese students, and the last one did not specify the country. This Special Issue is considered a significant contribution to the field of neuroeducation in physical activity and sport. The manuscripts are diverse in subject matter and design, which enriches the variety of the issue. The relevance of active teaching methodologies in the classroom is evident, as they allow the student to be involved throughout the process. Motor skills, approached from a pedagogical and integrative perspective, favor student learning exponentially, thus favoring their psycho-pedagogical and evolutionary development. Physical education teaching professionals need to reflect on the importance of connecting with the interests of their students, seeking and enhancing the transferability of learning.

## Data Availability

Not applicable.
